# Signaling Pathways Involved in Myocardial Ischemia–Reperfusion Injury and Cardioprotection: A Systematic Review of Transcriptomic Studies in *Sus scrofa*

**DOI:** 10.3390/jcdd9050132

**Published:** 2022-04-26

**Authors:** Hector Salazar-Gonzalez, Yanet Karina Gutierrez-Mercado, Francisco Javier Munguia-Galaviz, Raquel Echavarria

**Affiliations:** 1 Departamento de Procesos Tecnológicos e Industriales, ITESO, Tlaquepaque 45604, Jalisco, Mexico; hector.salazar@iteso.mx; 2 Departamento de Clinicas, Centro Universitario de los Altos, Universidad de Guadalajara, Tepatitlan 47620, Jalisco, Mexico; yanet.gutierrez@academicos.udg.mx; 3 Departamento de Fisiologia, Centro Universitario de Ciencias de la Salud, Universidad de Guadalajara, Guadalajara 44340, Jalisco, Mexico; francisco.munguia4072@alumnos.udg.mx; 4 Division de Ciencias de la Salud, Centro Universitario del Sur, Universidad de Guadalajara, Ciudad Guzman 49000, Jalisco, Mexico; 5 CONACYT-Centro de Investigacion Biomedica de Occidente, Instituto Mexicano del Seguro Social, Guadalajara 44340, Jalisco, Mexico

**Keywords:** myocardial ischemia, cardioprotection, transcriptomics, signaling pathways, RNA-seq, microarrays, swine

## Abstract

Myocardial damage in acute myocardial infarctions (AMI) is primarily the result of ischemia–reperfusion injury (IRI). Recognizing the timing of transcriptional events and their modulation by cardioprotective strategies is critical to address the pathophysiology of myocardial IRI. Despite the relevance of pigs for translational studies of AMI, only a few have identified how transcriptomic changes shape cellular signaling pathways in response to injury. We systematically reviewed transcriptomic studies of myocardial IRI and cardioprotection in *Sus scrofa*. Gene expression datasets were analyzed for significantly enriched terms using the Enrichr analysis tool, and statistically significant results (adjusted *p*-values of <0.05) for Signaling Pathways, Transcription Factors, Molecular Functions, and Biological Processes were compared between eligible studies to describe how these dynamic changes transform the myocardium from an injured and inflamed tissue into a scar. Then, we address how cardioprotective interventions distinctly modulate the myocardial transcriptome and discuss the implications of uncovering gene regulatory networks for cardiovascular pathologies and translational applications.

## 1. Introduction

Acute myocardial infarction (AMI), an ischemic injury that often results from coronary artery occlusion, is a leading cause of morbidity and mortality worldwide [1,2]. An insufficient oxygen supply leads to progressive changes in the coronary microcirculation and in the cardiomyocytes that irreversibly damage a significant portion of the myocardium [3,4]. During ischemia, the cellular metabolism shifts to anaerobic respiration, thus contributing to mitochondrial membrane depolarization, increased cytosolic calcium, ATP depletion, higher lactate production, cardiomyocyte death, and hindered myocardial contractile function [5]. Reperfusion restores the blood flow after ischemia, alleviating some ischemic damage. Nonetheless, reperfusion also initiates a harmful inflammatory response and subjects the myocardium to sudden biochemical and metabolic changes that further damage the infarct region [6]. Therefore, developing effective strategies to protect cardiomyocytes against ischemia–reperfusion injury (IRI) is vital for treating AMI and reducing its severe health and economic burdens.

Prompt myocardial reperfusion using thrombolytic therapy or primary percutaneous coronary intervention lessens myocardial infarct size, preserves left ventricular systolic function, and reduces the onset of heart failure, though with limited efficacy [7]. Moreover, preclinical studies promising mechanical, pharmacological, and molecular interventions against myocardial IRI have translated poorly into improved clinical outcomes in patients [8,9,10,11]. Rodents are the most often used species to study cardiovascular physiology and disease. However, these small animal models insufficiently represent human pathophysiological features and may not be reliable predictors of drug responses in pharmacological studies [12]. Thus, a significant challenge in developing effective cardioprotective strategies relies on preclinical models better representing myocardial failure and infarction. 

The pig (*Sus scrofa*) is a suitable species for biomedical research since it shares many similarities with humans, including body size, anatomical features, physiology, and pathophysiology [13]. The pig heart’s structure and fetal development also resemble those of humans [14]. Furthermore, preclinical models of heart disease have demonstrated the appropriateness of the pig for studies that require hemodynamic monitoring, myocardial behavior assessment, and imaging measurements, as they can generate data to develop algorithms able to guide medical interventions in human patients [15]. Additionally, the porcine genome is three times closer to humans than the mouse genome, and detailed analyses of the genes associated with human disease and drug–gene interactions have uncovered a substantial similarity between both species [16,17].

Studying gene expression datasets derived from microarrays and high-throughput sequencing can reveal novel functional interdependencies among signaling pathways that concertedly determine cellular responses [18,19]. Understanding the timing of transcriptional events and their modulation by cardioprotective strategies is critical to address the pathophysiology of myocardial IRI [20]. Despite the relevance of pigs for translational studies of AMI, only a few studies have identified the transcriptomic landscape of myocardial IRI and how changes in gene expression shape cellular signaling pathways in response to injury.

Here, we will review transcriptomic studies that use *Sus scrofa* as a preclinical model to gain insight into the signaling pathways involved in myocardial IRI and cardioprotection. First, we will integrate gene expression data from experimental studies found through a systematic search into signaling pathways. Then, we will present how cardioprotective interventions distinctly modulate the myocardial transcriptome. Finally, we will discuss the implications of uncovering gene regulatory networks for cardiovascular pathology and translational applications.

## 2. Materials and Methods

This systematic review is reported following the Preferred Reporting Items for Systematic Reviews and Meta-analyses (PRISMA) statement [21].

### 2.1. Elegibility Criteria

We selected experimental studies of myocardial IRI in *Sus scrofa* that included gene expression analysis using either microarrays or high-throughput sequencing. All studies in species other than the pig, non-original articles (reviews, editorials, and commentaries), transcriptomic studies focused solely on non-coding RNA, studies on single-cell RNA sequencing, and unpublished Gene Expression Omnibus (GEO) DataSets were excluded from the review. All eligible studies were grouped into two categories: (1) studies with transcriptomic data of myocardial IRI compared to healthy tissue and (2) studies with transcriptomic data of myocardium in response to any cardioprotective intervention, with or without IRI.

### 2.2. Search Strategy

Next, we searched the PubMed database (https://pubmed.ncbi.nlm.nih.gov/, accessed on 9 March 2022) and the GEO database repository (https://www.ncbi.nlm.nih.gov/geo/, accessed on 9 March 2022) starting from their inception and up to February 2022, without language limitation. Three independent searches were conducted in PubMed using the keywords “Myocardial ischemia reperfusion” AND “RNA sequencing”, “Myocardial ischemia reperfusion” AND “microarray”, “Left anterior descending coronary artery AND RNA sequencing”, and “Left anterior descending coronary artery” AND “microarray”. In addition, the keywords “Myocardial ischemia reperfusion” and “Left anterior descending coronary artery” were also introduced into the GEO database using the filters for species (*Sus scrofa*) and study type (expression profiling by array and expression profiling by high throughput sequencing). Selected articles contained gene lists or open access datasets of differentially expressed genes (DEGs) derived from microarray analysis or high-throughput sequencing.

Duplicate papers were removed. For the risk of bias assessment, three independent reviewers initially screened the title and abstract of each study. Then two reviewers assessed the full texts, excluded non-eligible studies, and confirmed eligible studies. Finally, any conflict regarding the studies was settled by consensus.

### 2.3. Data Collection

For each study, the following data were retrieved: first were the author, year of publication, swine breed, sex, age, surgical procedure, occlusion time, reperfusion time, area analyzed, type of cardioprotective intervention, transcriptomic platform, GEO accession number, and lists of DEGs. Two reviewers independently extracted the data, and discordance was settled by consensus. We extracted the DEGs (adjusted *p*-value < 0.05) from each publication (S1). When the DEGs could not be retrieved directly from the publication, we analyzed the open access GEO datasets using GEO2R (http://www.ncbi.nlm.nih.gov/geo/geo2r/, accessed on 11 March 2022) [22]. All the initial data manipulation was performed on Excel 2017, version 15.33 (Microsoft Corporation, Bellevue, WA, USA).

### 2.4. Enrichment Analysis

Gene expression data sets were analyzed for significantly enriched terms using the Enrichr analysis tool (https://maayanlab.cloud/Enrichr/, accessed on 11 March 2022) [23,24]. Statistically significant results (adjusted *p*-values of <0.05) for Signaling Pathways (SPs), Transcription Factors (TFs), Molecular Functions (MFs), and Biological Processes (BPs) (S2) were selected according to their relevance and compared between the included studies to identify similarities and discrepancies. Equivalent TFs, SPs, MFs, and BPs across datasets were manually curated. Only consistent results (i.e., from two or more data sets) were considered, clustered according to their histological region as an Infarct Core (IC), the area primarily affected by the ischemic insult that is infarcted or irreversibly condemned to infarct regardless of reperfusion, or Border Zone (BZ), the tissue usually located around the IC that is at risk of evolving to infarction but can still be rescued by reperfusion, and ordered chronologically. The authors in each of the publications reviewed determined how the IC and BZ were identified and processed for their transcriptomic study. To confirm the relevant results, we functionally analyzed them with the g: Profiler (https://biit.cs.ut.ee/gprofiler/gost, accessed on 11 March 2022) toolset using *Sus scrofa* as the organism to match the input query gene list [25]. All data on the remote zone post-IRI were excluded from the analysis.

### 2.5. Venn Diagrams

To identify comparisons in SPs and TFs from different models’ data sets, we graphed relevant Enrichment analysis results (adjusted *p*-values of <0.05) using the Venn diagrams online drawer tool (https://bioinformatics.psb.ugent.be/webtools/Venn/, accessed on 13 March 2022).

### 2.6. Reactome Pathway Analysis

DEGs in response to cardioprotective interventions were subjected to Reactome Pathway Analysis (https://reactome.org/PathwayBrowser/#TOOL=AT, accessed on 16 March 2022), and the 25 most relevant pathways (adjusted *p*-values of <0.05) were retrieved [26].

## 3. Results

**Figure 1:**
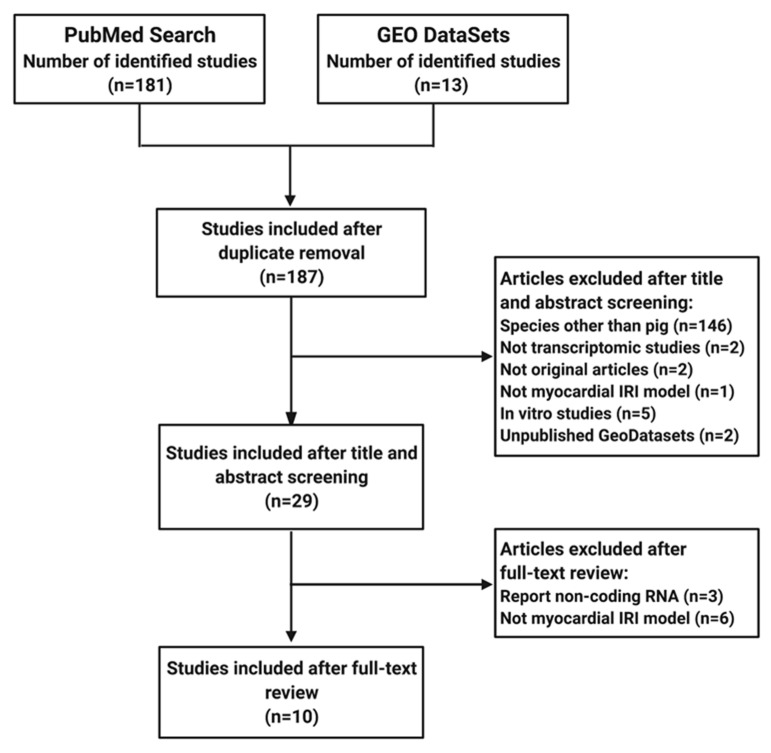
Diagram of the systematic review process.

A diagram of the systematic review process is presented in Figure 1. From the ten studies included, four contained transcriptomic data of early post-infarction myocardial tissue in the first (≤24 h) and second window (≥72 h). Another four studies addressed transcriptional modulation at later times during chronic ischemic heart failure (≥28 days). Only eight studies contained transcriptomic data in response to cardioprotective interventions. Table 1 summarizes the IRI models, the cardioprotective interventions, and the transcriptomic platforms from each publication.

### 3.1. Time Course of Transcriptional Events in Myocardial IRI

IRI starts immediately after reperfusion, unleashing dynamic biochemical and mechanical changes that transform the myocardium from an injured tissue into a scar by coordinating distinct healing phases (acute inflammation, repair, and chronic ischemic heart failure) [37]. Through enrichment analysis of DEGs, we found temporal and spatial changes in SPs, TFs, MFs, and BPs.

#### 3.1.1. Signaling Pathways Upregulated in the IC and the BZ

**Figure 2:**
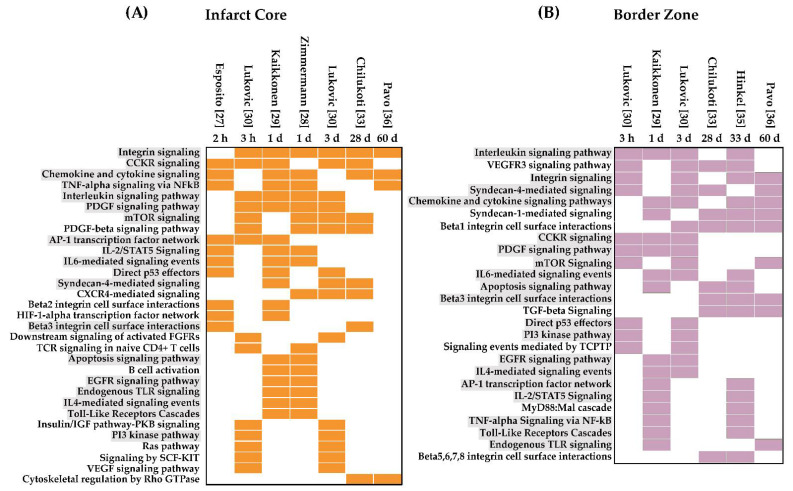
Active Signaling Pathways (SPs) in the Infarct Core (**A**) and the Border Zone (**B**). For each histological area, upregulated genes registered in the published data sets were analyzed separately using Enrichr and g: Profiler to identify active SPs. Only results with significant adjusted *p*-values (<0.05) were considered as active in the tissues corresponding to each data set. Identified active SPs in at least two data sets were defined as active in this comprehensive IRI analysis. Active SPs were ordered chronologically. Colored boxes indicate the active SPs. Active SPs common to the Infarct Core and the Border Zone are highlighted in grey. Listed Active SPs from each histological area were compared to identify similarities using a Venn diagram (not shown).

According to Gene Enrichment analysis, thirty-one pathways were upregulated in the IC after myocardial IRI (Figure 2A). Our analysis revealed that SPs activated by integrins, chemokines, cytokines, tumor necrosis factor (TNF)-alpha, platelet-derived growth factor (PDGF), and mTOR consistently appear two hours post-infarction and remain upregulated even after ≥28 days. At early time points post-IRI (≤3 days), DEGs were associated with the AP-1 transcription factor network, IL2/STAT5 signaling, IL6-mediated signaling events, HIF1A transcription factor network, and p53 effectors. However, transcriptomic changes at the IC were more pronounced after 1 day, when essential pathways for acute inflammation become activated. These pathways include T cell receptor (TCR) signaling, apoptosis, B cell activation, and toll-like receptor (TLR) cascades. None of these early pathways were upregulated at later time points (≥28 days). In contrast, cytoskeletal regulation by Rho GTPases was the only pathway enriched exclusively during the scar maturation phase in the IC. Then, when the upregulated DEGs were analyzed for outstanding outcomes of cell signaling, the results reflect many defining features of infarction and ischemia, including hypoxia, apoptosis, inflammation, angiogenesis, coagulation, and myocardial dysfunction.

In contrast, only twenty-six SPs were upregulated in the BZ after IRI (Figure 2B). Most pro-inflammatory signaling cascades appear as early as 3 h from the start of reperfusion and persist even after 28 days, suggesting that although the BZ suffers less damage than the IC, inflammation in the BZ is unresolved. Similarly, integrin signaling remained upregulated at different time points, indicating active tissue repair post-infarction. Among the enriched signals restricted to the early phase (up to 3 days), we found the EGFR, PDGF, PI3K, and p53 pathways, whereas transforming growth factor (TGF)-beta signaling activity occurs only at later stages (28–60 days).

Although we primarily focused on upregulated SPs for their translational potential to identify novel drug targets, we also performed enrichment analysis of downregulated DEGs. The biological significance of the enrichment analysis reflected the downregulation of SPs known to associate with ischemia and AMI, such as a reduced oxidative metabolism, calcium imbalance, and impaired cardiomyocyte contractile function (data not shown). In general, analyzed downregulated gene datasets were heterogenic; therefore, it was difficult to reach a clear consensus when comparing enrichment results.

Interestingly, we found nineteen SPs common to the IC and the BZ (Figure 2A,B) involved in inflammation, apoptosis, and extracellular matrix (ECM) remodeling. Undoubtedly, the striking discrepancies between the progression and fate of both areas must rely upon differences in the signaling events’ intensity, timing, specificity, and regulation. It is essential to notice the apparent divergence between the outcomes’ timelines in the IC and the BZ (S2). While the pathological processes in the IC appeared to be temporarily scattered and activated in just a few hours post-infarction, in the BZ, the significant outcomes were mainly restricted to the chronic phase (28, 33, and 60 days). Pathways associated with ECM remodeling and myogenesis were among the main events happening late at the BZ, whereas only hypoxia and angiogenesis appeared as active processes throughout all recorded times.

#### 3.1.2. Transcription Factors Upregulated in Myocardial IRI

**Figure 3:**
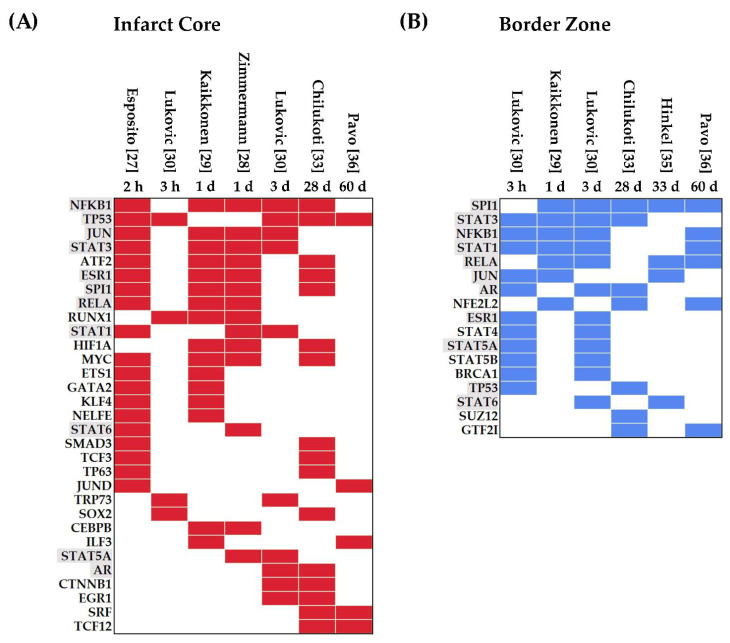
Active Transcription Factors (TFs) in the Infarct Core (**A**) and the Border Zone (**B**). For each histological area, upregulated genes registered in the published data sets were analyzed separately using Enrichr to identify active TFs in each study. Only results with significant adjusted *p*-values (<0.05) were considered as active in the tissues corresponding to each data set. Identified active TFs in at least two data sets were defined as active in this comprehensive IRI analysis. Active TFs were ordered chronologically. Colored boxes indicate the active TFs. Active TFs common to the Infarct Core and the Border Zone are highlighted in grey. Listed Active TFs from each histological area were compared to identify similarities using a Venn diagram (not shown).

TFs regulate cellular processes and provide a link between signaling pathways and gene regulation [38]. Here, we identified thirty-one TFs in the IC (Figure 3A). Nuclear factor-kappa b (NFKB1) and TP53 were the most frequently upregulated TFs, present in five out of seven data sets analyzed. NFKB1 upregulation started at the early phases of myocardial IRI, and its expression was maintained up to 28 days post-infarction. Similarly, TP53’s upregulation appeared 2 h after the start of reperfusion and remained increased at 60 days.

In the IC, several TFs were differentially expressed during the first and second windows post-infarction (≤3 days) including JUN, STAT3, ATF2, ESR1, SPI1, RELA, RUNX1, STAT1, MYC, ETS1, GATA2, KLF4, NELFE, and STAT6 (Figure 3A). Many of these early response TFs are cardioprotective and mediate DNA damage responses, metabolic regulation, immunity, and inflammation [39,40,41]. In contrast, the AR, CTNNB1, and EGR1 were identified as upregulated TFs throughout the acute and chronic stages (≥3 days), which correlates with their role in modulating transcriptional programs associated with resolution and repair in response to tissue injury [42,43,44]. Additionally, ubiquitous nuclear proteins such as SRF and basic helix–loop–helix transcription factors such as TCF12 play crucial functions in cell fate specification and differentiation during cardiac development [45,46]. Both SRF and TCF12 were upregulated at 28 and 60 days, highlighting their involvement during the chronic stages of myocardial IRI.

In the BZ, we found seventeen upregulated TFs predominantly associated with inflammation and proliferation (Figure 3B). Of note is that several members of the STAT family were identified at times ranging from 3 h up to 60 days. In addition, NFE2L2, a modulator of oxidative stress, was upregulated at 1, 28, and 60 days [47,48]. Finally, eleven TFs were upregulated in datasets from both areas (IC and BZ), namely, SPI1, STAT1, STAT3, STAT5A, STAT6, NFKB1, RELA, AR, ESR1, JUN, and TP53 (Figure 3A,B).

#### 3.1.3. Molecular Functions and Biological Processes in Myocardial IRI

**Figure 4:**
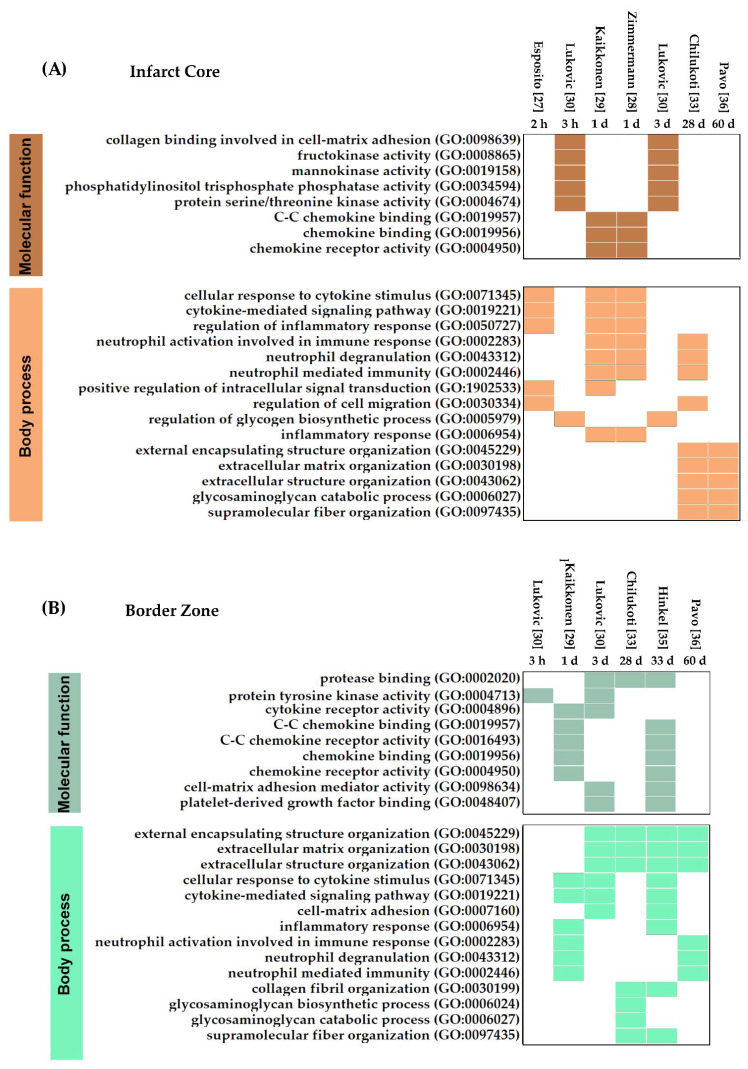
Active molecular functions and body processes in the Infarct Core (**A**) and the Border Zone (**B**). For each histological area, upregulated genes registered in the published data sets were analyzed separately using Enrichr and g: Profiler to identify active MFs and BPs in each study. Only results with significant adjusted *p*-values (<0.05) were considered as active in the tissues corresponding to each data set. Identified active MFs and BPs in at least two data sets were defined as active in this comprehensive IRI analysis. Functions and Processes were ordered chronologically. Colored boxes indicate the active Functions and Processes.

Analysis of DEGs at the IC identified chemokine binding, CC chemokine binding, and chemokine receptor activity as the most prominent MFs 1-day post-infarction (Figure 4A). This strong chemokine response is a characteristic of the inflammatory response in reperfused myocardial infarction and may play an essential role in regulating leukocyte recruitment, angiogenesis, and fibroid tissue deposition [49]. In addition, we found early and sustained responses up to 3 days after IRI related to the carbohydrate metabolism (mannokinase and fructokinase), collagen binding in cell–matrix adhesion, and kinase and phosphatase activities.

Most of the early processes (≥1 day) transcriptionally activated after myocardial IRI correlated with the hallmarks of the inflammatory response (i.e., cytokine stimulus, cell migration, neutrophil degranulation, and modulation of the immune response) (Figure 4B). Although neutrophil activity was also detected at 28 days post-infarction, the prominence of inflammation diminishes at later times. Instead, the most notable changes detected during the chronic phase of myocardial IRI were related to ECM organization and turnover. Thus, the first days after IRI are defined by an intense inflammatory response in the IC that fades progressively. After a month, the main biological processes are related to changes in the ECM responsible for the ventricular remodeling and the scar formation occurring at chronic stages.

In the BZ, the MFs found at early time points were consistent with the inflammatory process and tissue remodeling found in the IC (Figure 4A,B). Some of these MFs, including chemokine activity, cytokine receptor activity, cell–matrix activity, and PDGF binding, remained detectable in the BZ even after 33 days post-infarction. Comparison between MFs’ profiles showed that chemokine-mediated responses occur in both the IC and BZ (Figure 4A,B), evidencing that leukocyte trafficking is a common phenomenon in both histological areas. The BPs occurring in the BZ are driven by ECM remodeling and inflammation (Figure 4B). Despite the similarities in the BPs’ analysis between the IC and BZ, it is important to distinguish that in the BZ, most of the processes occur during the chronic phase of IRI (28, 33, and 60 days), reflecting a delay in the onset of the processes.

### 3.2. Gene Expression Profiles Induced by Cardioprotective Strategies

Cardioprotection encompasses all actions and interventions aimed at reducing myocardial IRI. However, of all the studies included, only eight contained information regarding DEGs in response to cardioprotective strategies (Table 1), and their findings are discussed below.

#### 3.2.1. Ischemic Preconditioning

Ischemic preconditioning (IPC) is a term initially coined by Murry CE et al. to describe the ability of short periods of ischemia to limit the infarct size that has widened to include beneficial effects on other IRI outcomes such as myocardial stunning and arrhythmias [50]. Depre C et al. have explored the transcriptomic profile of three experimental swine models of IPC that mimic the clinical conditions encountered by patients who often experience repetitive episodes of ischemia and reduce the infarct size 60–85% [32].

**Figure 5:**
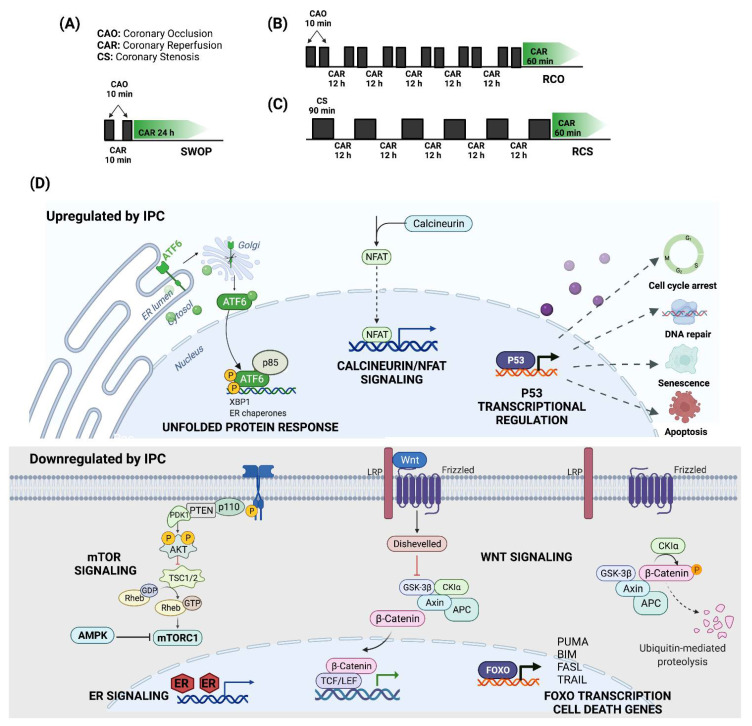
Cell signaling pathways modulated by IPC. IPC models (**A**) SWOP; (**B**) RCO; (**C**) RCS; (**D**) Main cardioprotective pathways up and downregulated in all three models of IPC, highlighting their widespread cardioprotective potential in myocardial IRI.

The first model, a classical second-window IPC (SWOP) that consists of 10-min episodes of coronary artery occlusion (CAO) followed by 24 h of reperfusion, depends on nitric oxide to exert its cardioprotective effects since pretreatment with a nitric oxide synthase (NOS) inhibitor abates the IPC (Figure 5A). In contrast, the other two IPC models, repetitive CAO/reperfusion (RCO) and repetitive coronary stenosis (RCS), elicit their cardioprotective effects independently of nitric oxide (Figure 5B,C). Microarray analysis revealed distinct transcriptional programs in response to each IPC model [32].

Furthermore, enrichment analysis of upregulated genes in SWOP showed pathways involved in mitochondrial translation, respiratory electron transport, ATP synthesis, glucose metabolism, organelle maintenance, p53-regulated transcription of genes involved in cell cycle arrest, and DNA damage recognition in global genomic nucleotide excision repairing (GG-NER) (Table 2). In contrast, the downregulated pathways included TGF-β receptor signaling via SMAD proteins, cytokine signaling, RUNX2 migration, and nuclear receptor transcriptional pathways (Figure 5D).

In the RCO model, immune pathways such as interferon-gamma signaling, class I MHC mediated antigen processing and presentation, Fcgamma receptor-dependent phagocytosis, and cytokine signaling were highly enriched, along with retinoic acid signaling and neddylation (Table 2). In addition, downregulated DEGs are mainly enriched in signaling by Rho GTPases, cell cycle, and transcription. Meanwhile, the RCS model induced upregulation of genes enriched in pathways associated with the cellular response to starvation and SLIT/ROBO signaling; and downregulation of genes involved in metabolism, adherens junctions’ interactions, and NFkB activation (Table 2).

Interestingly, enrichment analysis demonstrates that a subset of DEGs is common in all three IPC models (SWOP, RCO, and RCS), suggesting that their influence on common pathways can act as a broad cardioprotective signature (Table 2). These enriched upregulated pathways include the unfolded protein response, metabolism of nucleotides, calcineurin activation of NFAT, transcriptional regulation by small RNAs, transcriptional regulation by p53, neutrophil degranulation, and mRNA splicing. Meanwhile, common downregulated pathways comprise AKT phosphorylation of its nuclear targets, WNT signaling, FOXO-mediated transcription of cell death genes, mTORC1-mediated signaling, interleukin signaling, and estrogen-dependent nuclear events (Table 2).

Shen Y et al. used the previously described IPC protocols (RCS and SWOP) in a swine model of lethal ischemia induced by 60 min of coronary artery occlusion followed by reperfusion to study transcriptional changes occurring at the subendocardium of the area at risk at 4 days post-infarction [31]. Only 31% of DEGs in this IRI model after RCS were also regulated in SWOP. Broad categories of genes induced by RCS but not SWOP included those involved in autophagy, endoplasmic reticulum stress. Thus, upregulation of genes in autophagy, ER stress, cell cycle, and cell survival, together with downregulation of genes in mitochondrial function, define the cardioprotective mechanisms elicited by the RCS model and distinguishes it from the SWOP model 4 days after myocardial IRI.

#### 3.2.2. Ischemic Postconditioning

Ischemic postconditioning (IPostC) consists of cycles of brief coronary occlusion-reperfusion applied during reperfusion soon after a sustained coronary occlusion that, unlike IPC, can be applied in patients undergoing interventional coronary reperfusion by primary Percutaneous Coronary Intervention for acute ST-segment elevation myocardial infarction (STEMI) [51]. In STEMI patients, IPostC limits infarct size, reduces microvascular obstruction, decreases edema, and improves contractile function [52]. In a clinically relevant porcine model, Lukovic D et al. have demonstrated that six 30 s cycles of occlusion-reperfusion applied immediately after a prolonged ischemic insult fails to reduce myocardial necrosis size but closely replicates the cardioprotective effect of IPostC on the coronary microvasculature seen on STEMI patients [30]. Furthermore, RNA-seq analyses on myocardial samples from pigs that underwent myocardial IRI followed by IPostC identified distinct DEGs in the IC. Enriched upregulated pathways included signaling by receptor tyrosine kinases (ERBB2 and ALK), MAPK family signaling cascades, intracellular signaling by second messengers, syndecan and integrin cell surface interactions (Table 3). In contrast, downregulated pathways associated with VEGFR2 mediated vascular permeability, energy dependent regulation of mTOR by LKB1-AMPK, regulation of p53 activity, and FLT3, FGFR1, SCF-KIT, and G-CSF signaling (Table 3).

#### 3.2.3. Primary LV Unloading

Percutaneously delivered transvalvular axial-flow pumps (TV-pump) are routinely used in the clinic to increase systemic mean arterial pressure while reducing left ventricular (LV) wall stress and myocardial oxygen demand [53]. Primary unloading the left ventricle using a TV-pump while delaying coronary reperfusion reduces the myocardial infarct size by more than half when compared to immediate reperfusion [54]. In a swine model of myocardial IRI, primary unloading decreases LV scar size and is associated with a higher stroke volume, cardiac output, and stroke work 28 days after injury [27]. The cardioprotective effects of primary unloading have mainly been attributed to an increase in the level and activity of the stromal-cell-derived CXCL12 [55]. Esposito ML et al. found that primary unloading increases circulating CXCL12 levels during the 28 days after myocardial IRI, which peaks at week one [27]. Moreover, LV unloading prior to reperfusion maintained the levels of this chemokine high within the IC.

Whole-transcriptome expression analysis on the infarct zone after the acute phase revealed that LV unloading for 30 min before reperfusion causes global gene expression changes and attenuates the transcriptomic response caused by reperfusion alone [27]. In addition, LV unloading regulates genes associated with the metabolism, mitochondrial function, and cellular respiration (Table 3). Enrichment analysis showed the upregulation of DEGs in the citric acid cycle and respiratory electron transport, mitochondrial protein import, cardiac conduction, mitochondrial fatty acid beta-oxidation, amino acid catabolism, p53 regulation of metabolic genes, neddylation, and muscle contraction. In contrast, downregulated DEGs belonged to pathways associated with ECM remodeling, non-integrin membrane–ECM interactions, integrin cell surface interactions, complement activation, regulation of IGF signaling, post-translational protein phosphorylation, notch signaling, platelet activation, semaphorin interactions, and RUNX2 regulation of myeloid cells’ differentiation.

#### 3.2.4. Pharmacological Cardioprotection

Many drugs assessed in preclinical studies for their potential effects on AMI target previously identified signal transduction pathways to either inhibit deleterious processes such as apoptosis and oxidative stress or promote cardioprotection via the increased formation of adenosine or nitric oxide [56,57,58]. Dronedarone is an antiarrhythmic drug that reduces cardiovascular mortality and the incidence of acute coronary syndromes in patients with Atrial Fibrillation [59]. Dronedarone also reduces the infarct size in animal models of acute myocardial IRI and cerebral infarction [60,61].

Chilukoti RK et al. orally administered dronedarone to pigs twice a day, starting 7 days before the experimental myocardial IRI and continuing after 28 days [33]. Although the hemodynamic parameters and infarct size remained unchanged by dronedarone, expression profiling on the IC and the BZ showed that dronedarone modifies the transcriptional response of myocardial IRI. Interestingly, dronedarone’s impact on gene expression occurs primarily at the BZ. Several pathways are significantly affected by dronedarone under conditions of AMI, including ephrin receptor signaling, hepatic fibrosis signaling, PKA signaling, adherence junction signaling, integrin signaling, inhibition of matrix metalloproteases, and cell death (Table 3).

In contrast, only minor changes in gene expression (NPPA, ELN, EFNB2, ACOX3, and GATA3) occur within the IC after dronedarone treatment. However, dronedarone was able to modify the intensity of the late myocardial IRI transcriptional response through significant modulation of ephrin receptor signaling, PKA signaling, adherence junction signaling, integrin signaling, mitochondrial dysfunction, and NFAT in cardiac hypertrophy (Table 3).

#### 3.2.5. AntagomiRs

MicroRNAs (miRNAs) are non-coding functional transcripts of around 22 nucleotides in length that post-transcriptionally regulate entire biological pathways [62]. miR-21 is critical during the early phase of AMI and is upregulated in both the left ventricular myocardium’s remote and border regions [63]. Recently, Hinkle R et al. studied the therapeutic efficacy of intracoronary delivery of a locked nucleic-acid-modified antimiR-21 (LNA-antimiR-21) in pigs that underwent transient percutaneous occlusion of their left coronary artery [35].

At 33 days after IRI, LNA-antimiR-21 reduced the infarct size and improved cardiac function. Moreover, RNA-seq analysis revealed a suppression of the inflammatory response and mitogen-activated protein kinase signaling [35]. Enrichment analysis of DEGs in the LNA-antimiR-21-treated BZ showed an upregulation of signal transduction mediators, particularly those associated with angiogenesis, such as Tie-2 signaling and netrin-mediated repulsion signals (Table 2). LNA-antimiR-21-induced cardioprotection was accompanied mainly by cardiac downregulation of genes belonging to signaling by interleukins and cytokines, FGFR2 ligand binding and activation, PI3K/AKT signaling, and WNT signaling.

#### 3.2.6. Regenerative Therapies

The adult heart is a highly specialized organ with limited regenerative potential in response to injury [64]. Hence, regenerative medicine therapies have aimed to repair damaged hearts by either directly replacing injured myocardial cells with contractile and noncontractile cells or by paracrinally modulating endogenous repair processes such as inflammation, apoptosis, angiogenesis, and fibrosis [65]. Pavo N. et al. have demonstrated that the secretome of apoptotic peripheral blood cells (APOSEC), which contains a combination of cytokines and growth factors, regenerates the myocardium after acute and chronic IRI [66]. Moreover, since APOSEC is derived from large numbers of readily-obtainable peripheral blood cells, it overcomes some of the inherent obstacles of cell therapy related to the relatively small number of available autologous adult cells compared to the large volume of cells required for intramyocardial delivery [67].

Percutaneous intramyocardial delivery of APOSEC at day 30 was safe and effective in a porcine model of chronic left ventricular dysfunction induced by myocardial IRI [36]. Moreover, at day 60 post-infarction, APOSEC-treated animals had significantly smaller infarcts, improved hemodynamic function, and enhanced vascular density compared to medium-treated animals. APOSEC’s cardioprotective effects coincided with DEGs in both IC and BZ. Only a few genes with known functions were upregulated in APOSEC-treated myocardium (TPM3, KLF11, MYOZ1, PRNP, and GNPAT). In contrast, enrichment analysis (Table 3) showed the downregulation of pathways associated with cytokine signaling, T cell receptor (TCR) signaling, p53 transcriptional regulation of cell death genes, inflammasome activation, and pyroptosis.

In a different study, Agnew EJ et al. addressed cardiac regeneration using a transient cardiac injury approach in weaned pigs at postnatal day (P) 30 when cardiomyocyte mitotic activity is still observed [34]. Nevertheless, there was no myocardial regeneration at 4 weeks after IRI, and only decreased cardiac function, scar formation, and increased inflammation were detected. RNA-seq analysis comparing the IC with healthy myocardium showed upregulation of CD69, CD72, CD86, CD209, IL10, IL18, IL18R, CXCL9, CXCL10, CXCL11, and CXCR3. Most transcriptional changes were associated with immune and inflammatory pathways and were confined to the scar zone rather than the BZ. Despite these findings, cardiac regeneration after IRI in young pigs appeared to be blocked by scar development. Moreover, the presence of mitotic activity in a subset of cardiomyocytes was not cardioprotective since it failed to promote cardiac repair, at least in this model of myocardial IRI.

## 4. Discussion

Much of our understanding of myocardial IRI’s pathology and cardioprotective interventions comes from preclinical acute and chronic tissue damage models. Even though none of the preclinical models perfectly recreates the cellular and molecular aspects found in humans, the use of animals such as the pig in biomedical research makes it possible to recapitulate diseases with a particular affinity in order to investigate their mechanisms and possible pharmacological and cellular treatments in less time than what would typically happen [12]. Furthermore, transcriptomic studies in porcine models of cardiovascular disease offer great potential for discovering novel pathogenic mechanisms and producing more significant results that can efficiently be applied to benefit human health [68].

**Figure 6:**
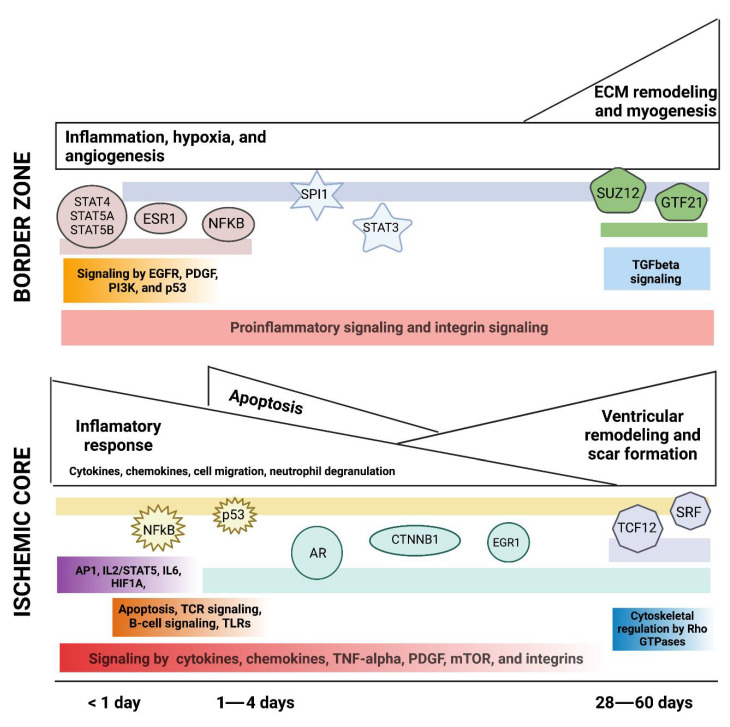
Spatiotemporal pathways transcriptionally modulated after myocardial IRI in swine.

Here, the integration of transcriptomic data from different studies of myocardial IRI provides us with an integrated view of the signaling complexities occurring in distinct cardiac areas (IC vs. BZ) and their dynamic modulation throughout time (Figure 6). In the IC, inflammation, immune cell infiltration, and apoptosis predominate during the first hours and days after reperfusion, gradually fading until being replaced by ECM protein accumulation and scarring, which alter the physical arrangement and stiffness of the tissue. Meanwhile, in the BZ, signaling pathways associated with inflammation, hypoxia, and angiogenesis that become upregulated soon after reperfusion remain present at chronic phases, even when ECM remodeling and myogenesis processes prevail. Many of the pathways upregulated after IRI have been previously described in other non-human, preclinical experimental models [20]. However, a direct comparison of the transcriptomic regulation of myocardial IRI between the pig and other animal models has not been addressed, and further studies are needed to uncover any distinct differences between animal models.

Despite the insights gained through the enrichment analyses of DEGs post-infarction, the transcriptomic studies reviewed here only considered early (≤4 days) and late (≥28 days) time points, thus leaving a gap in between corresponding to the phase in which the resolution of inflammation and tissue repair occurs [37]. There are significant translational implications of elucidating the gene regulatory networks preceding late adverse remodeling events characterized by the progressive cross-linking of collagen and elastin fibers that make scar resolution increasingly challenging [69]. Understanding how signaling pathways that influence post-IRI repair are transcriptionally modulated could lead to identifying potential therapeutic targets that may successfully influence cardiac regeneration rather than repair. Furthermore, when overstimulated, the same pathways that promote regeneration progressively drive scarring and tissue decay due to damage-induced ECM deposition [70]. Thus, transcriptomic studies of myocardial IRI in swine at the repair and resolution phase are urgently needed to uncover time-dependent gene networks at the intersection between regeneration and fibrosis.

Cardioprotective interventions in the experimental models of myocardial IRI reviewed prevented cell death during acute injury and attenuated the destructive processes that occur during ventricular remodeling through the downregulation of oxidative stress, inflammatory pathways (interleukins, cytokines, myeloid cells differentiation, TCR signaling), ECM remodeling, FGFR2 signaling, and p53 activity (Table 3). Although an excessive and sustained inflammatory response post-IRI leads to increased cell death, adverse remodeling, and contractile dysfunction, there is a lack of successful therapeutic strategies targeting pro-inflammatory signaling pathways. Several clinical and preclinical studies targeting inflammation through the inhibition of complement cascades, interleukins, and matrix metalloproteinases hold promise for major adverse clinical event reductions in patients with AMI [71]. However, the complexity of the resolution of inflammation and the healing process makes it necessary to search for novel cardioprotective pathways that can be modulated to improve AMI outcomes.

**Figure 7:**
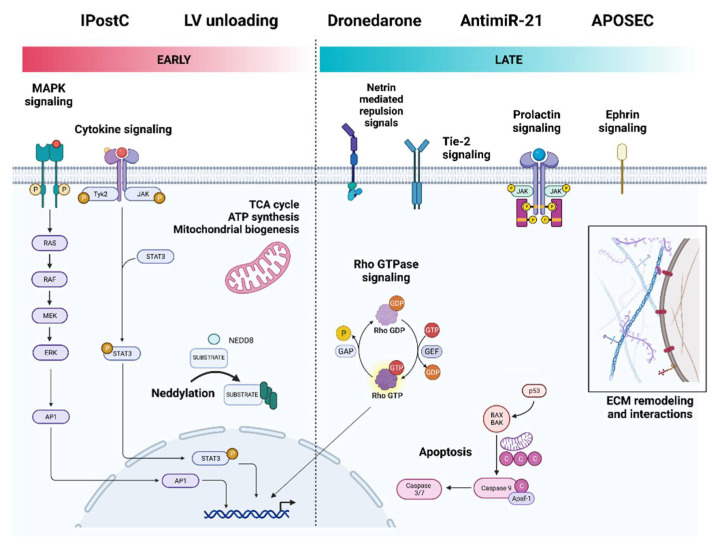
Cardioprotective pathways in myocardial IRI.

Transcriptomic analysis of cardioprotective interventions early post-infarction (Table 3) revealed the upregulation of MAPK cascades, the mitochondrial energy metabolism, and neddylation (Figure 7). Various studies have established a link between MAPK signaling and mitochondria [72]. Mitochondrial KATP channels protect against myocardial IRI, and cardioprotective strategies that activate PKC also potentiate mitochondrial KATP channel opening [73]. Moreover, the opening of mitochondrial KATP channels activates p38 MAPK, whereas anisomycin, a MAPK activator, is cardioprotective, and this effect is blocked by mitochondrial KATP channels’ inhibition [74]. Additionally, pharmacological modulation after myocardial IRI to increase the cardiac energy metabolism has been proposed to manage ischemic damage from AMI [75]. Multiple approaches to enzymatic machinery inhibition have been tested to reduce the rate of fatty acid oxidation, switch the source of acetyl-CoA to pyruvate derived from glucose, glycogen, and lactate, generate more significant amounts of ATP, reduce the harmful effects of fatty acid metabolites, and decrease lactate and H^+^ production during ischemia and reperfusion.

Neddylation, a recently described protein conjugation pathway like the ubiquitin-proteasome system, is deregulated in patients suffering from dilated and ischemic cardiomyopathy [76]. Neddylation promotes cardiomyocyte survival and regulates autophagy under oxidative stress conditions [77]. Moreover, inhibiting neddylation using MLN4924 limits the infarct size after IRI, thus suggesting that deficient ubiquitination–proteasome coupling contributes to myocardial IRI [78].

In contrast, ephrin signaling, Tie2 cascades, netrin-mediated repulsion signals, and Rho GTPase signaling have emerged as upregulated pathways during the chronic phase in response to cardioprotective interventions (Figure 7). Ephrin ligands are classified into two subclasses: EphrinA ligands anchored to the cell membrane by a glycosyl-phosphatidyl-inositol linkage and transmembrane-spanned EphrinB ligands. The interaction between Ephrin ligands and their Eph receptors has been proposed as a potential therapeutic target in AMI treatment, particularly EphrinA1-Fc, that influences cardiomyocyte survival and regeneration [79,80].

The endothelial Tie2 receptor tyrosine kinases, together with the angiopoietins, belong to an endothelial-specific signaling pathway with essential functions in the regulation of cardiovascular development and vascular homeostasis [81]. Tie2 gene-targeted mouse embryos are embryonically lethal and exhibit impaired cardiac development [82]. Interestingly, angiopoietin-1 prevents vascular leakage, promotes cardiomyocyte survival via integrin-β1-mediated ERK phosphorylation, and improves hemodynamic parameters after myocardial IRI [83].

Additionally, netrins belong to a family of laminin-like proteins initially described in axonal guidance [84]. Netrin-1 regulates angiogenesis in response to ischemic insults and exerts a cardioprotective effect in myocardial infarctions via ERK1/2-dependent nitric oxide activation by endothelial nitric oxide synthase (eNOS) [85]. Moreover, small netrin-1-derived peptides are highly effective in protecting the heart against myocardial IRI and have been proposed as drugs directly applicable to the treatment of myocardial infarctions [86].

Finally, the Ras homolog gene family member A (RhoA) from the Rho GTPase superfamily controls actin dynamics, signal transduction, and transcription, thus affecting survival, proliferation, and migration [87]. RhoA is essential for cardiac remodeling, and its cardiac-specific overexpression results in dilated cardiomyopathy and heart failure [88]. However, Rho GTPase signaling has also been described as cardioprotective by modulating the target genes implicated in cardiomyocyte differentiation, cell growth, proliferation, and anti-apoptotic signaling pathways. One of these genes is CCN1, a growth-factor-inducible early gene associated with proliferation and survival signaling in cardiomyocytes and SRF [89]. Additionally, Rho cardioprotective mechanisms have been linked to the activation of PTEN and PLC, leading to increased cytoplasmic Ca^2+^ levels to promote cardiomyocyte contractility [90]. Furthermore, the RhoA/ROCK-induced activation of PI3K has been shown to promote cell survival signaling by activating AKT signaling [91].

In this systematic review, our analysis of transcriptomic changes in experimental swine models of myocardial IRI was limited by the small number of studies, the fact that many publications failed to provide complete data on DEGs, the sample size, the heterogeneity of transcriptomic platforms, the lack of consideration of non-coding RNAs, and the vast array of cardioprotective interventions. Additionally, the included studies exhibited considerable differences in their subject characteristics (different swine breed, age, sex, and hormonal status) and experimental conditions (ischemia and reperfusion times). Moreover, systematic reviews, followed by meta-analysis, are urgently needed to analyze and combine results from similar transcriptomic studies to further understand gene expression changes and cell signaling pathways elicited by IRI and cardioprotection.

## 5. Conclusions

Transcriptomic analyses of myocardial IRI in swine highlight spatiotemporally controlled signaling pathways that recapitulate the events of inflammation, cell recruitment, apoptosis, and ECM deposition that are characteristic of this lesion. Increasing our knowledge of gene regulatory networks modulated by cardioprotective interventions in suitable preclinical models of myocardial IRI can lead to novel therapeutic targets for AMI. Although the striking heterogeneity of cardiac remodeling poses a significant challenge for the clinical implementation of cardioprotective interventions, a coordinated attempt is necessary to elucidate the molecular signals responsible for adverse remodeling. Furthermore, understanding cell signaling’s transcriptomic modulation balancing tissue regeneration and fibrosis is essential to developing effective therapeutic interventions and predicting disease progression.

## Figures and Tables

**Table 1:** Summary of studies included in the systematic review.

References	Breed	Sex	Occlusion/Reperfusion	Area	Intervention	Platform	GEO Accession
Esposito ML et al., 2018 [27] *	Yorkshire	Male	90 min/120 min	IC	LV unloading	Porcine 1.0 ST microarrays (Affymetrix)	GSE108644
Zimmer-mann M et al., 2017 [28] *	Domestic	Female	90 min/1 d	IC/BZ/RZ	-	Whole Porcine Genome Oligo Microarray (Agilent)	-
Kaikkonen MU et al., 2017 [29] *	Farm	Female	90 min/1 d	IC/BZ	-	GRO-SeqIllumina HiSeq2000	GSE81155
Lukovic D et al., 2019 [30] *	Domestic	Female	90 min/3 h 90 min/3 d	IC/RZ	IPostC	RNA-SeqIllumina HiSeq2500	-
Shen YT et al., 2008 [31]	Unspecified	Unspecified	60 min/4 d	SE	IPC	Porcine Genome Array (Affymetrix)	-
Depre C et al., 2010 [32]	Domestic	Female	10 min Twice/1 d 10 min Twicex6/1 h	Heart	IPC	Porcine Genome Array (Affymetrix)	GSE21096
Chilukoti RK et al., 2018 [33] *	German Landrace	Male, castrated	90 min/28 d	IC/BZ	Dronedarone	GeneChip Porcine Genome Arrays (Affymetrix)	-
Agnew EJ et al., 2019 [34]	Yorkshire Landrace	Male/Female	60 min/30 d	LV	Age	RNA-SeqIllumina NovaSeq6000	GSE137293
Hinkel R et al., 2020 [35] *	German Landrace	Male/Female	60 min/33 d	BZ	LNA-antimiR-21	RNA-SeqIllumina HiSeq2000	-
Pavo N et al., 2014 [36] *	Domestic	Female	90 min/60 d	IC/BZ	APOSEC	*Sus Scrofa* Oligo Microarray v2 (Agilent)	GSE47397

* Studies of myocardial IRI with DEGs used for enrichment analysis. Abbreviations: GEO, Gene Expression Omnibus; IC, ischemic core; BZ, border zone; RZ, remote zone; SB, subendocardium; LV, left ventricle; IPostC, ischemic postconditioning; IPC, ischemic preconditioning; LNA, locked nucleic acid; miR, microRNA; APOSEC, secretome of apoptotic peripheral blood cells.

**Table 2:** Pathways modified by IPC interventions in the heart.

IPC Model	Pathway Name	
	Upregulated	Downregulated
RCO	Interferon gamma signaling Class I MHC antigen presentation Signaling by retinoic acid Cytokine Signaling in the immune system FCGR activation Fcγ receptor dependent phagocytosis Chromosome maintenance Neddylation	Cell cycle Gene expression (Transcription) mRNA 3’-end processing RNA Pol II transcription termination Transport of transcript to cytoplasm mRNA splicing Signaling by Rho GTPases
RCS	Nonsense Mediated Decay (NMD) independent of the Exon Junction Complex (EJC) SRP-dependent cotranslational protein targeting to membrane Cellular response to starvation Eukaryotic Translation Elongation Response of EIF2AK4 (GCN2) to amino acid deficiency Major pathway of rRNA processing in the nucleolus and cytosol Selenocysteine synthesis Signaling by SLIT/ROBO receptors Neutrophil degranulation Signaling by Interleukins	Respiratory electron transport Pyruvate metabolism and citric acid (TCA) cycle Mitochondrial biogenesis and protein import Glyoxylate metabolism and glycine degradation TFAP2 (AP-2) family regulates transcription of growth factors and their receptors Triglyceride catabolism RUNX1 and FOXP3 control of Tregs Hormone ligand-binding receptors Striated muscle contraction Adherens junctions’ interactions TRAF6 and TAK1 mediated NF-kB activation Gluconeogenesis Myogenesis
SWOP	Translation Respiratory electron transport and ATP synthesis Organelle biogenesis and maintenance Insertion of tail-anchored proteins into the endoplasmic reticulum membrane Gluconeogenesis Glucose metabolism Nectin/Necl trans heterodimerization Class I peroxisomal membrane protein import TP53 regulates transcription of genes involved in G2 cell cycle arrest DNA Damage Recognition in GG-NER	TGF-beta receptor signaling activates SMADs Chemokine receptors bind chemokines Response to elevated platelet cytosolic Ca^2+^ RHO GTPases activate CIT Nuclear receptor transcription pathway Interleukin-10 signaling Platelet activation, signaling, and aggregation Response of EIF2AK1 (HRI) to heme deficiency RUNX2 regulates genes involved in cell migration Signaling by interleukins Signaling by GPCR
RCP/RCS/SWOP	Unfolded Protein Response (UPR) XBP1(S) and IRE1alpha activate chaperone genes Pyrophosphate hydrolysis Metabolism of nucleotides Calcineurin activates NFAT Transcriptional regulation by small RNAs ATF6 (ATF6-alpha) activates chaperone genes TP53 regulates transcription of DNA repair genes Translation Neutrophil degranulation Protein repair mRNA Splicing—minor pathway	RUNX3 regulates BCL2L11 (BIM) transcription AMPK inhibits chREBP transcriptional activation activity AKT phosphorylates targets in the nucleus mTORC1-mediated signaling WNT mediated activation of DVL Defective binding of RB1 mutants to E2F1, (E2F2, E2F3) FOXO-mediated transcription of cell death genes Deadenylation of mRNA Interleukin-4 and Interleukin-13 signaling Estrogen-dependent nuclear events Beta-oxidation of pristanoyl-CoA

**Table 3:** Pathways modified by cardioprotective interventions after myocardial IRI.

Cardioprotective Intervention	Pathway Name	
	Upregulated	Downregulated
IPostC (3 d)	Cytokine signaling in the immune system Signaling by receptor tyrosine kinases Syndecan interactions Intracellular signaling by second messengers Signaling by ALK MAPK family signaling cascades Integrin cell surface interactions Signaling by ERBB2	Detoxification of reactive oxygen species MTOR signaling Signaling by SCF-KIT Cytokine signaling in the immune system Signaling by CSF3 (G-CSF) and FGFR1 Energy-dependent regulation of mTOR by LKB1-AMPK VEGFR2-mediated vascular permeability FLT3 signaling Regulation of TP53 activity
LV unloading (2 h)	Citric acid cycle and respiratory electron transport Complex I biogenesis Cristae formation and mitochondrial protein import Muscle contraction Cardiac conduction Mitochondrial fatty acid beta-oxidation Branched-chain amino acid catabolism TP53 Regulates Metabolic Genes Mitochondrial translation Neddylation Regulation of pyruvate dehydrogenase complex	ECM organization and degradation Integrin cell surface interactions Activation of C3 and C5 Regulation of insulin-like growth factor Post-translational protein phosphorylation Non-integrin membrane-ECM interactions Platelet activation, signaling and aggregation Signaling by NOTCH3 Other semaphorin interactions RUNX2 regulates genes involved in differentiation of myeloid cells
Dronedarone (28 d)	ECM organization and degradation Syndecan interactions Signaling by MET Integrin cell surface interactions Smooth Muscle Contraction Non-integrin membrane–ECM interactions CD163 mediating an anti-inflammatory response SMAC, XIAP-regulated apoptotic response Apoptosome-mediated caspase activation EPH-Ephrin signaling	Mitochondrial translation Citric acid cycle and respiratory electron transport Ketone body metabolism Branched-chain amino acid catabolism Pyrophosphate hydrolysis Formation of TC–NER pre-incision complex RHO GTPases activate CIT Nuclear Receptor transcription pathway
LNA-antimiR-21 (33 d)	Tie2 Signaling MAPK1/MAPK3 signaling STAT5 activation Netrin mediated repulsion signals Signaling by Leptin Prolactin receptor signaling Interleukin-6 signaling	Chemokine receptors bind chemokines Signaling by Interleukins and cytokines Negative regulation of FGFR2 signaling FGFR2 ligand binding and activation Phospholipase C-mediated cascade; FGFR2 Negative regulation of TCF-dependent signaling by WNT ligand antagonists PI3K/AKT Signaling
APOSEC (60 d)	Plasmalogen biosynthesis Striated muscle contraction NCAM1 interactions Peroxisomal protein import Glycerophospholipid biosynthesis Phospholipid metabolism RHO GTPase cycle Signaling by Rho GTPases	Cytokine signaling and cell recruitment TP53 regulates transcription of cell death genes Pyroptosis PD-1 signaling DAP12 interactions The AIM2/IPAF inflammasome Nef and signal transduction TCR signaling

## Data Availability

The data presented in this study are available as supplementary material.

## Supplementary Materials

The following supporting information can be downloaded at: https://www.mdpi.com/article/10.3390/jcdd9050132/s1, Supplementary File S1.xls: The full list of DEGs from each publication; Suppplementary File S2.xls: The full list of Enrichment analyses.
